# Effects of *Aedes aegypti* salivary components on dendritic cell and lymphocyte biology

**DOI:** 10.1186/1756-3305-6-329

**Published:** 2013-11-15

**Authors:** Bruna Bizzarro, Michele S Barros, Ceres Maciel, Daniele I Gueroni, Ciro N Lino, Júlia Campopiano, Michalis Kotsyfakis, Gustavo P Amarante-Mendes, Eric Calvo, Margareth L Capurro, Anderson Sá-Nunes

**Affiliations:** 1 Laboratório de Imunologia Experimental, Departamento de Imunologia, Instituto de Ciências Biomédicas, Universidade de São Paulo, São Paulo, 05508-900, SP, Brazil; 2 Laboratório de Biologia Celular e Molecular, Departamento de Imunologia, Instituto de Ciências Biomédicas, Universidade de São Paulo, São Paulo, SP 05508-900, Brazil; 3 Laboratory of Genomics and Proteomics of Disease Vectors, Institute of Parasitology, Biology Centre of the Academy of Sciences of Czech Republic, Ceske Budejovice 37005, Czech Republic; 4 Instituto de Investigação em Imunologia, Instituto Nacional de Ciência e Tecnologia, INCT, São Paulo, Brazil; 5 Section of Vector Biology, Laboratory of Malaria and Vector Research, National Institute of Allergy and Infectious Diseases, National Institutes of Health, Rockville, MD 20852, USA; 6 Laboratório de Mosquitos Geneticamente Modificados, Departamento de Parasitologia, Instituto de Ciências Biomédicas, Universidade de São Paulo, São Paulo, SP 05508-900, Brazil; 7 Instituto Nacional de Ciência e Tecnologia em Entomologia Molecular, Conselho Nacional de Desenvolvimento Científico e Tecnológico (INCT-EM/CNPq), Rio de Janeiro, Brazil

**Keywords:** Dendritic cells, T cells, *Aedes aegypti*, Saliva, Apoptosis

## Abstract

**Background:**

Saliva is a key element of interaction between hematophagous mosquitoes and their vertebrate hosts. In addition to allowing a successful blood meal by neutralizing or delaying hemostatic responses, the salivary cocktail is also able to modulate the effector mechanisms of host immune responses facilitating, in turn, the transmission of several types of microorganisms. Understanding how the mosquito uses its salivary components to circumvent host immunity might help to clarify the mechanisms of transmission of such pathogens and disease establishment.

**Methods:**

Flow cytometry was used to evaluate if increasing concentrations of *A. aegypti* salivary gland extract (SGE) affects bone marrow-derived DC differentiation and maturation. Lymphocyte proliferation in the presence of SGE was estimated by a colorimetric assay. Western blot and Annexin V staining assays were used to assess apoptosis in these cells. Naïve and memory cells from mosquito-bite exposed mice or OVA-immunized mice and their respective controls were analyzed by flow cytometry.

**Results:**

Concentration-response curves were employed to evaluate *A. aegypti* SGE effects on DC and lymphocyte biology. DCs differentiation from bone marrow precursors, their maturation and function were not directly affected by *A. aegypti* SGE (concentrations ranging from 2.5 to 40 μg/mL). On the other hand, lymphocytes were very sensitive to the salivary components and died in the presence of *A. aegypti* SGE, even at concentrations as low as 0.1 μg/mL. In addition, *A. aegypti* SGE was shown to induce apoptosis in all lymphocyte populations evaluated (CD4^+^ and CD8^+^ T cells, and B cells) through a mechanism involving caspase-3 and caspase-8, but not Bim. By using different approaches to generate memory cells, we were able to verify that these cells are resistant to SGE effects.

**Conclusion:**

Our results show that lymphocytes, and not DCs, are the primary target of *A. aegypti* salivary components. In the presence of *A. aegypti* SGE, naïve lymphocyte populations die by apoptosis in a caspase-3- and caspase-8-dependent pathway, while memory cells are selectively more resistant to its effects. The present work contributes to elucidate the activities of *A. aegypti* salivary molecules on the antigen presenting cell-lymphocyte axis and in the biology of these cells.

## Background

Mosquitoes are the most important vectors of human pathogens, transmitting a wide number of emerging and re-emerging diseases. In particular, *Aedes aegypti* mosquitoes are the primary vectors of yellow fever, dengue fever and Chikungunya fever [1-4]. The key interaction element between *A. aegypti* and its vertebrate host is the mosquito saliva and a successful blood meal is achieved by the action of salivary anti-hemostatic and immunomodulatory molecules present in this pharmacological cocktail. The former are responsible for anticoagulant, anti-platelet aggregation and vasodilatory activities [5,6], while the latter is thought to modulate immune functions, which in turn, facilitates pathogen transmission. Indeed, a growing number of recent pieces of evidence have demonstrated that *A. aegypti* salivary components increase viral infection *in vitro* and *in vivo*[7-12] and an increased reactivity with mosquito salivary proteins is observed in sera from infected individuals, suggesting a positive correlation between mosquito exposure and risk of infection [13,14].

As a result of their strategic location in the skin, dendritic cells (DCs) interact directly with the mosquito salivary components during and after the blood meal. They are also among the first cells to encounter pathogens transmitted by these vectors and initiate the adaptive immune response against them. Following this initial contact, DCs mature and migrate to the draining lymph nodes, becoming effective stimulators of T cell responses [15]. Despite DCs essential role in connecting innate and adaptive immune responses, very little is known about the effects of *A. aegypti* salivary components on these cells. A previous study has demonstrated that *A. aegypti* salivary gland extract (SGE) does not affect the viability or IL-12 production by a fetal skin-derived DC line (FSDC) [16]. Therefore, *A. aegypti* SGE has no effect on the basal expression of IFN-β by DCs, but it decreases the production of this cytokine in the presence of West Nile Virus infection [9]. In addition to its putative effects on DCs, *A. aegypti* SGE was shown to affect the proliferation of murine lymphocytes *in vitro*[16-18] and inhibit the production of proinflammatory (GM-CSF and TNF-α) and Th1 cytokines (IL-2 and IFN-γ), but modest effects were observed on Th2 cytokines levels (IL-4 and IL-5) [16]. On the other hand, it has been shown that the salivary protein SAAG-4 induces CD4^+^ T cells to express IL-4 [19]. Accordingly, splenocytes from mice previously exposed to *A. aegypti* bites produced higher levels of IL-4 and IL-10 and decreased IFN-γ production [20]. Additionally, recent literature has demonstrated an important functional relationship between coagulation and immunity [21-23] and, in fact, some of the salivary anti-hemostatic molecules described in hematophagous arthropods are also involved in the modulation of host inflammation and immune responses through different mechanisms and pathways [20,24-26].

However, despite the effects described above and the mosquitoes relevance as disease vectors, the immunomodulatory activities of *A. aegypti* saliva on the antigen presenting cell-lymphocyte axis is still very limited. In the current study, we examined the activity of *A. aegypti* SGE on several parameters of DC and lymphocyte biology. Employing murine cells, we demonstrated that modulation of DC maturation, differentiation or function does not seem to be a priority for *A. aegypti* salivary components. Conversely, direct inhibition of naïve T cell proliferation caused by apoptosis is already achieved with low amounts of *A. aegypti* SGE, through a mechanism involving cleavage of pro-caspase-3 and pro-caspase-8, but not the proapoptotic Bcl-2 homolog Bim. Interestingly, memory cells generated by different approaches are selectively resistant to this activity.

## Methods

### Mice

All the experiments were carried out in accordance with internationally recognized guidelines and approved by the Animal Care of the Institute of Biomedical Sciences of University of São Paulo (CEUA-ICB/USP) and under the protocol number 91/2009. Female BALB/c, C57BL/6, DO11.10 (expressing a TCR transgenic for the sequence of OVA 323–339), Bim^+/−^ and Bim^−/−^ (a proapoptotic member of the Bcl-2 family) mice at 6–16 weeks old were bred and maintained at the Department of Immunology, ICB/USP, Brazil.

### Mosquitoes and sandflies

Male and female *A. aegypti* and *Anopheles aquasalis* mosquitoes were reared in an insectary at the Department of Parasitology, ICB/USP, Brazil. Temperature was maintained at 26°C, 80% humidity and a 12/12-h photoperiod. Larvae were fed on powdered fish food and adult mosquitoes were given continuous access to a 10% sucrose solution. Salivary glands from *Phlebotomus duboscqi* sandflies were kindly provided by Dr. Jesus G. Valenzuela, from the Vector Molecular Biology Section, Laboratory of Malaria and Vector Research, National Institutes of Allergy and Infectious Diseases, National Institutes of Health (LMVR/NIAID/NIH, USA).

### Salivary gland extracts

Female mosquitoes aged 4–6 days had their surface sterilized by brief immersion in 70% ethanol, prior to dissection. The salivary glands were dissected in PBS and transferred to a microtube containing 50 μL of cold PBS. Salivary glands of female *P. duboscqi* were collected under the same conditions described above. The tubes containing salivary glands were sonicated to release the soluble material and centrifuged at 14,000 *g* for 10 minutes to remove particulate material. The resulting supernatant, referred to as SGE, was collected and sterilized by passage through a nitrocellulose membrane filter with 0.2 μm pores (Millipore, Carrigtwohill, County Cork, Ireland). Protein concentration was determined by NanoDrop 2000 (Thermo Fisher Scientific, Wilmington, DE, USA) and aliquots were stored at −80°C until use.

### Differentiation and maturation of bone marrow-derived DCs (BMDCs)

BMDCs were employed as a model to study DC biology. Bone marrow cells collected from the femurs of mice were cultured in complete medium (RPMI 1640 medium with 10% heat-inactivated FBS, 2 mM L-glutamine, 100 U/mL penicillin, 100 μg/mL streptomycin, 25 mM Hepes, 2.5 × 10^5^ M 2-mercaptoethanol) and 20 ng/mL of murine GM-CSF to induce DC differentiation [24,27]. In order to define if the salivary components would affect the differentiation process, cells were incubated (37°C and 5% CO_2_) in aliquots of 1 mL in sterile 24 well plates in the presence of medium only or *A. aegypti* SGE at 2.5, 5, 10, 20 and 40 μg/mL concentrations (6 replicates per group). After 4 days of incubation, non-adherent cells from three replicates of each group were collected by washing with complete medium. On the three remaining replicates, half the volume was removed and replaced by an equal volume of complete medium again containing 40 ng/mL GM-CSF in the presence or absence of the same concentrations of SGE. At 7 days of incubation, non-adherent cells of the remaining three replicates were collected as described above. In both cases, cells were counted and differentiation of bone marrow cells into DCs in different culture times was assessed by flow cytometry to evaluate the expression of CD11b and CD11c surface markers.

For the maturation assays, BMDCs were obtained and differentiated for 6 days with GM-CSF as described above. Non-adherent cells were collected, resuspended at 10^6^ cells/mL and distributed into sterile 24 well plates in aliquots of 1 mL per well. These cells were incubated overnight with medium only or medium containing SGE. Then, cells were stimulated with ultrapure LPS (100 ng/mL - final concentration) for 24 h to promote their maturation and then labeled with antibodies to MHC class II, CD40, CD80 and CD86 [24,27].

### Antigen presentation by DCs

In order to evaluate the antigen presentation by BMDCs, non-adherent cells were collected at 7 days of incubation and CD11c^+^ cells were purified using magnetic MACS columns (Miltenyi Biotec Inc., Auburn, CA, USA). In brief, BMDC suspensions were incubated with anti-CD11c MicroBeads (Miltenyi Biotec Inc.) for 15 min at 4°C, followed by a PBS wash, and then sorted using MACS columns (MS). Analysis of the sorted cells showed purity of 80–90% CD11c^+^ cells (data not shown). CD11c^+^ cells were preincubated overnight (37°C and 5% CO_2_) with medium or SGE at various concentrations (2.5, 5, 10, 20 and 40 μg/mL). After this period, cells were incubated for 4 h in the presence of LPS (100 ng/mL) plus OVA (100 μg/mL). After three washes, 2.5 × 10^5^ cells/mL were distributed in aliquots of 100 μL per well into a 96-well plate. CD4^+^ T lymphocytes from DO11.10 mice (which express a specific transgenic TCR for the OVA peptide 323–339) were purified using magnetic MACS columns. A suspension containing 10^6^ cells/mL was prepared and 100 μL were added to wells on the culture plate (DC:lymphocyte ratio 1:4) and maintained at 37°C and 5% CO2 for 72 h.

Similar experiments were performed, but after DC washing, *A. aegypti* SGE was added again in the culture, followed by addition of CD4^+^ T cells from DO11.10 mice (that express a specific transgenic TCR for the OVA peptide 323–339). Cells were stimulated with OVA (100 μg/mL) plus LPS (100 ng/mL) or Con A (0.5 μg/mL). In the last 24 h of the culture, 25 μL of 0.01% resazurin were added to all wells. Cell proliferation was evaluated by reading the culture absorbance at 570 and 600 nm, and results are expressed as the difference between those readings as previously described [24,27,28].

### Spleen cell proliferation

Following euthanasia, spleens of BALB/c mice were aseptically removed and a cell suspension containing 10^6^ cells/mL was prepared. Cells were then divided into aliquots of 100 μL/well in 96-well plates. Next, 50 μl of *A. aegypti*, *An. aquasalis* or *P. duboscqi* at various concentrations were added in the culture and incubated for 30 min. After pre-incubation, 50 μL of Con A was added to each well (0.5 μg/mL – final concentration), and cultures were incubated for 72 h at 37°C and 5% CO_2_. Proliferation was evaluated as described [24,27,28].

### Apoptosis

To assess the proportion of viable cells and ‘debris’, spleen cells were incubated in the presence of *A. aegypti* SGE (final concentration 10 μg/mL) or medium for 72 h. After this period, the percentage of viable cells was evaluated by flow cytometry using the forward scatter (cell size) and side scatter (granularity) parameters and compared to fresh cells.

For apoptosis quantification, spleen cells were incubated in the presence of *A. aegypti* SGE or medium for 4, 8, 24 and 72 h. Cells were labeled with lymphocyte markers (CD4, CD8 and CD19) for subpopulation analysis. Cells were then washed twice with PBS and resuspended with 500 μL of Annexin-binding buffer (0.1 M Hepes, 1.4 M NaCl, 25 mM CaCl_2_). Subsequently, 5 μL of Annexin V-FITC was added to the cell suspension, which was then incubated in the dark for 10 min at room temperature. Cells were evaluated by flow cytometry and apoptosis was measured by the percentage of Annexin V^+^ cells.

For Western blot analysis, spleen cells from BALB/c mice were prepared as described previously. Cell suspensions containing 20 × 10^6^ cells/ml were incubated with medium only or in the presence of *Ae. aegypti* SGE (final concentration: 10 μg/mL) and stimulated with Con A (final concentration 0.5 μg/mL) for 4 h at 37°C and 5% CO_2_. Cells were then collected, washed twice with PBS and lysed with 100 μL of RIPA buffer (150 mM NaCl, 1% NP40, 0.1% SDS, 50 mM Tris; pH 8.0) supplemented with 1% of protease inhibitor (Sigma- Aldrich, St. Louis, MO, USA). The lysate supernatant was collected after 10 minutes incubation, centrifuged at 14000 *g* for 5 minutes and the protein concentration was determined using BCA Protein Assay Kit according to the manufacturer's instructions (Thermo Fisher Scientific, Rockford, IL, USA).

The equivalent of 30 μg protein from each sample were diluted v/v in Laemmli buffer (2% SDS, 10% glycerol, 1% β -mercaptoethanol, 0.02% bromophenol blue, 0.4 M Tris), heated to 100°C for 5 minutes and separated by electrophoresis in a 12% Sodium Dodecyl Sulfate (SDS) polyacrylamide gel under a constant current of 20 mA. The separated proteins were transferred to a nitrocellulose membrane which was then blocked for 1 hour with 5% FBS diluted in Tris buffer containing 0.1% Tween −20 (TBST). Membranes were washed 3 times with TBST (10 minutes per wash) and incubated overnight at 4°C with the following rabbit monoclonal antibodies: anti-pro-caspase-3 (1:500) and anti-pro-caspase-8 (1:1,000), both from Cell Signaling Technology, Inc. (Danvers, MA, USA). After further washing, the membranes were incubated for 1 hour at room temperature with anti-rabbit secondary antibodies (1:2,000) conjugated with horseradish peroxidase for detection (Dako, Glostrup, Denmark). Immunoreactive bands were stained using the chemiluminescent ECL Detection Kit (Thermo Fisher Scientific) and visualized in a photodocumentation system. The membranes were then washed and incubated overnight with anti β-actin (1:10,000), followed by another washing and subsequent incubation with a anti-mouse secondary antibody (1:60,000). The density of the bands was analyzed using ImageJ software (available free at http://rsb.info.nih.gov/nih-image). The values were normalized by the total of β-actin present in each sample and expressed as arbitrary units.

### Mice sensitization by mosquito bite

Male and female mosquitoes *A. aegypti* and *An. aquasalis* were kept in plastic containers of about 12 cm diameter covered with a fine mesh screen. Mice were anesthetized and placed on the screen for 20–30 min so that female mosquitoes had direct contact with the animal’s skin for enough time to complete the blood meal, as previously standardized (data not published). This whole procedure was performed four times at 15 day intervals, using approximately 50 female mosquitoes per mice and the proportion of mosquitoes fed at each sensitization was typically ≥ 80%. Mice from the control group were also anesthetized, but had no contact with mosquitoes. Fifteen days after the last sensitization, proliferation was performed as described above.

### Adoptive transfer and mice immunization

Spleen cells from DO11.10 mice were prepared in complete medium and incubated at 37°C and 5% CO_2_ for 2 h in Petri dishes. Non-adherent cells were collected and the cell suspension was prepared in PBS containing 1% fetal bovine serum. These cells were inoculated i.v. to BALB/c mice in a volume of 200 μL containing 3 × 10^6^ cells. After 24 h, these mice were immunized subcutaneously at two sites of the dorsal region with 100 μL of an emulsion containing OVA and complete Freund's adjuvant v/v (40 μg of OVA per animal). After 7 days, spleens cells of control and immunized animals were collected and cell suspensions (10^6^ cells/mL) prepared. *In vitro* re-stimulation assays were performed with cells pre-incubated with medium alone or SGE for 30 minutes followed by stimulation with OVA (100 μg/mL) or Con A (0.5 μg/mL). The proliferation of these cells was assessed as described above. Naïve and memory phenotyping was performed in CD4^+^ T cells by flow cytometry as follows: CD62L^HIGH^/CD44^LOW^ (naïve cells), CD62L^HIGH^/CD44^HIGH^ (T_CM_ cells), and CD62L^LOW^/CD44^HIGH^ (T_EM_ cells).

### Flow cytometry

Cells were prepared for flow cytometry as previously described [24,27]. Fluorochrome-conjugated monoclonal antibodies against the following molecules were employed: CD4-APC, CD19-PE, CD11b-FITC, CD11c-PE, CD40-FITC, CD80-PerCP-Cy5.5, CD86-PE-Cy7 (BD Biosciences, San Jose, CA); CD8-PerCP, Annexin-FITC, I-A/I-E-APC, CD44-Pacific Blue, CD62L-PE (Biolegend, San Diego, CA, USA). Cells were acquired in a FACSCanto II (BD Biosciences) and analysis was performed using FlowJo software, version 7.5.5 (Tree Star, Ashland, OR, USA).

### Statistical analysis

Statistical analysis of differences between means of experimental groups was performed using Student’s *t* test (for two groups comparisons) or analysis of variance (ANOVA) followed by Tukey as a post-test (for three or more groups). A value of *p* ≤ 0.05 was considered statistically significant.

## Results

### *A. aegypti* SGE does not interfere with dendritic cell differentiation or maturation

**Figure 1:**
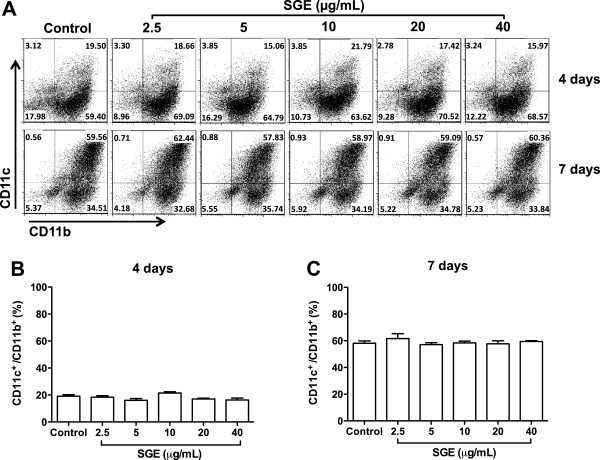
***A. aegypti *SGE does not interfere with DC differentiation.** BMDC from BALB/c mice were cultured with GM-CSF in the presence or absence of *A. aegypti* SGE (final concentration: 2.5, 5, 10, 20 and 40 μg/mL) for 4 and 7 days and analyzed by flow cytometry. Dot plots **(A)** and the mean of relative percentage of CD11b^+^/CD11c^+^ cells present in culture at 4 days **(B)** and 7 days **(C)** are presented.

BMDCs from BALB/c mice were cultured with GM-CSF in the presence of different concentrations of *A. aegypti* saliva and their differentiation was evaluated by flow cytometry through the percentage of CD11c^+^/CD11b^+^ cells. Figure 1 shows that *A. aegypti* SGE did not affect the differentiation of BMDCs at 4 or 7 days of culture (Figure 1A), as comparable percentages of CD11c^+^/CD11b^+^ cells were observed in all groups on both days (Figures 1B and 1C). These sets of experiments were also performed with BMDC from C57BL/6 mice and similar results were achieved (data not shown).

**Figure 2:**
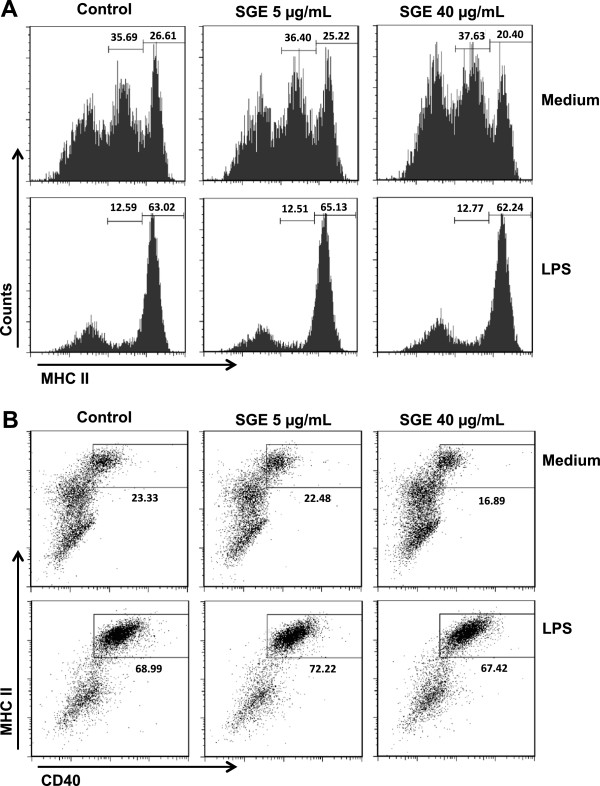
**DC maturation is not affected by *A. aegypti *SGE.** DCs were differentiated with GM-CSF for 6 days, preincubated overnight in the presence or absence of *A. aegypti* SGE (final concentration: 5 and 40 μg/mL) and stimulated or not with LPS (100 ng/mL), as indicated. Expression of MHC class II **(A)** and CD40 **(B)** was evaluated in CD11c^+^ cells by flow cytometry.

To assess whether *A. aegypti* saliva has an effect on DCs maturation, we analyzed the expression pattern of MHC class II and costimulatory molecules on DCs after incubation with LPS in the presence of SGE. As expected, immature DCs present variable levels of extracellular MHC class II expression (low, mid and high) and, upon LPS stimulation, the majority of these cells shift to MHC class II^HIGH^, confirming DC maturation. However, the presence of SGE did not alter the expression of MHC class II either in immature or mature DCs (Figure 2A). No differences were found in the sample’s mean fluorescent intensity (MFI), indicating similar levels of expression of this marker (data not shown). The expression of CD40 was also upregulated in CD11c^+^ cells upon LPS stimulation when compared to control cells (incubated with medium only), but again, the presence of SGE in the culture did not affect CD40 expression (Figure 2B). The same was observed with CD80 and CD86 expression (data not shown).

### *A. aegypti* SGE inhibits T cell proliferation in a DC-independent fashion

**Figure 3:**
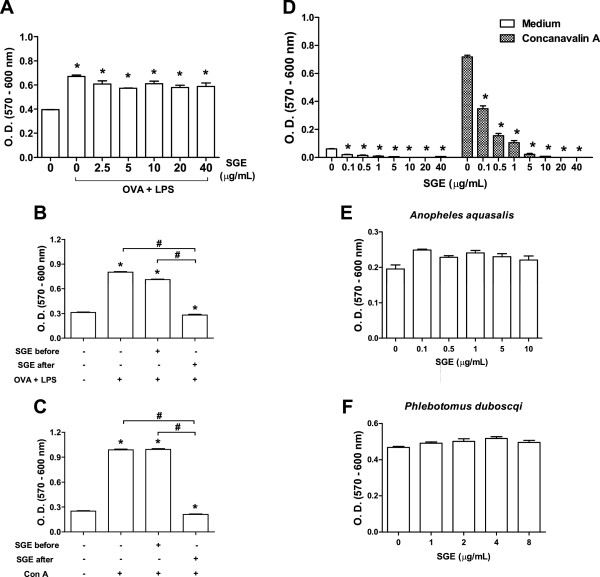
***A. aegypti *SGE inhibits T cell proliferation in a DC**-**independent fashion.** DCs were pre-incubated overnight in the presence or absence of *A. aegypti* SGE (final concentrations indicated) and stimulated for 4 h with OVA (100 μg/mL) plus LPS (100 ng/mL). After 3 washings, DCs were co-incubated with CD4^+^ cells from DO11.10 mice for 72 h **(A and B)**. Similar DC/CD4^+^ cultures were also stimulated by Con A for 72 h **(C)**. In some groups, SGE was added again after washing the cells **(B and C)**. Concentration-response effect of *A. aegypti* SGE on Con A-induced spleen cells proliferation **(D)**. Absence of effect of *An. aquasalis ***(E)** and *P. dubosqi* SGE **(F)** on Con A-induced spleen cells proliferation. ^*^p < 0.05 *versus* “C” (control); ^#^*p* < 0.05 *versus* stimulated with OVA + LPS or Con A.

In order to investigate the effect of *A. aegypti* SGE on antigen presentation by DCs, purified CD11c^+^ cells were incubated with SGE, pulsed with OVA plus LPS and after repeated washings to remove SGE, OVA and LPS residues, these cells were coincubated with CD4^+^ T lymphocytes from DO11.10 mice and the proliferation was evaluated [24,27,28]. OVA-pulsed DCs stimulated CD4^+^ T cell proliferation when compared to control (DCs incubated with medium alone). Nonetheless, when DCs were preincubated with *A. aegypti* SGE, the specific proliferation of CD4^+^ T cells was not affected (Figure 3A).

In another set of experiments, SGE was added again to the culture after DC washing, followed by coincubation with T cells from DO11.10 mice. Under these conditions, antigen-specific CD4^+^ T cell proliferation was completely abolished (Figure 3B). The same approach was performed, but stimulating the cultures with Con A, a polyclonal activator of T cells. Figure 3C shows that if SGE is maintained in the culture, polyclonal activation of T cells is also completely inhibited. These results were confirmed through CFSE staining (Additional file 1). Together, these data show that *A. aegypti* salivary components affect T lymphocytes in a DC-independent manner.

Next, we evaluated the potency of this inhibitory activity by incubating spleen cells with increasing concentrations of SGE followed by Con A stimulation. Figure 3D shows that SGE induced a concentration-dependent decrease of T cell proliferation, reaching maximal inhibition at 10 μg/mL. Of note, similar inhibitory activity was found for spleen cells from C57BL/6 mice (data not shown). We have also tested if salivary components of hematophagous species other than *A. aegypti* possessed such inhibitory activity. Interestingly, neither SGE from *An. aquasalis* mosquitoes (Figure 3E) nor from *P. duboscqi* sandflies (Figure 3F) were able to affect lymphocyte proliferation under the same conditions.

### *A. aegypti* SGE induces caspase-3 and caspase-8 dependent lymphocyte apoptosis

**Figure 4:**
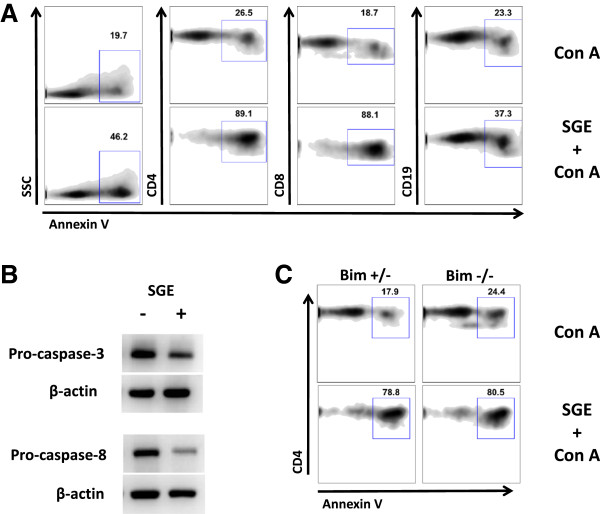
***A. aegypti *SGE induces lymphocyte apoptosis and cleavage of pro**-**caspase 3 and pro**-**caspase**-**8.** Total spleen cells were incubated with Con A in medium only or in presence of *A. aegypti* SGE for 4 h. Annexin V staining was evaluated by flow cytometry in total cells, CD4^+^, CD8^+^ and CD19^+^ cells from WT mice **(A)** or CD4^+^ T cells from Bim^+/−^ and Bim^−/−^ mice **(C)**. Lysates of similar cell cultures were blotted against a monoclonal antibody against pro-caspase-3 and pro-caspase-8 **(B)**.

As expected, when a spleen cell culture stimulated with Con A for 3 days is analyzed by flow cytometry, most lymphocytes appear larger in “size” and with more granularity compared to fresh cultures, indicating their activation (Additional file 2). The presence of *A. aegypti* SGE in this culture drastically changes cell phenotype, as the amount of “viable cells” is inversely proportional to SGE concentration, suggesting cell death (Additional file 2). In order to investigate the mechanism by which cells would be dying in the presence of SGE, we evaluated several apoptosis parameters. Figure 4A shows that annexin V staining is increased in total spleen cells stimulated by Con A in the presence of *A. aegypti* SGE when compared to cells stimulated with Con A only. A similar phenotype is observed in CD4^+^ and CD8^+^ T cells population as well as in CD19^+^ B cells (Figure 4A).

In order to further characterize the molecular pathway(s) involved in the apoptosis induced by *A. aegypti* salivary components, we evaluated the expression of pro-caspase-3 and pro-caspase-8 in total spleen cell lysates. As observed in Figure 4B, levels of both pro-caspases are reduced in cells incubated with Con A plus SGE when compared to cells stimulated with Con A alone.

### Naïve and memory T cells are differentially affected by *A. aegypti* SGE

Memory cells are known to be more resistant to apoptosis than naïve T cells [29]. Because Bim, a proapoptotic member of the Bcl-2 family, is involved in T cell homeostasis and memory T cells are resistant to Bim-induced apoptosis [30], we tested whether spleen cells from Bim knockout mice (Bim^−/−^) were as sensitive as their heterozygous counterparts (Bim^+/−^) to the inhibitory effects of *A. aegypti* SGE. Figure 4C shows that spleen cells from either Bim^−/−^ or Bim^+/−^ are equally affected by SGE, suggesting that the intrinsic apoptosis pathway does not play a role in SGE-induced apoptosis.

**Figure 5:**
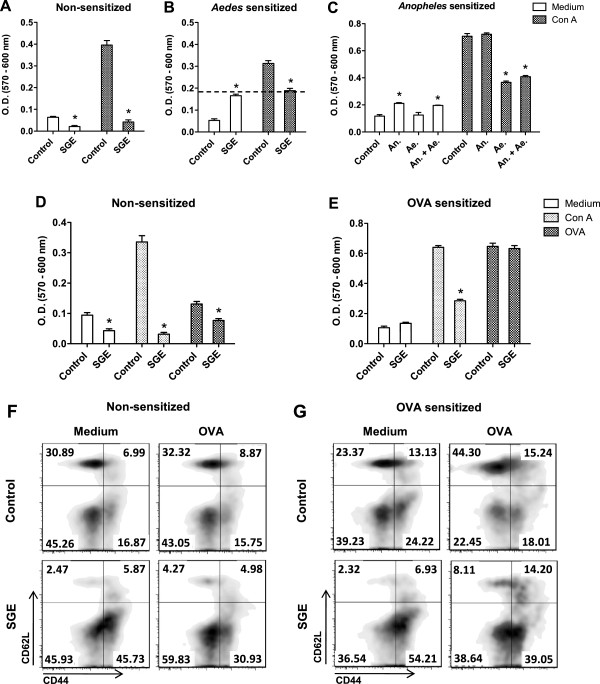
**Memory cells are resistant to *A. aegypti *SGE effects.** Spleen cells from non-sensitized **(A)** and *A. aegypti*-sensitized BALB/c mice **(B)** were incubated with medium only or with *A. aegypti* SGE (5 μg/mL) and/or stimulated with Con A (0.5 μg/mL) for 72 h. Cells from *An. aquasalis*-sensitized mice were cultured with medium only or in the presence of 5 μg/mL *An. aquasalis* SGE (An.) and/or 5 μg/mL *A. aegypti* SGE (Ae.) and then stimulated with 0.5 μg/mL Con A **(C)**. Non-adherent DO11.10 spleen cells were adoptively transferred to BALB/c mice and after 7 days, recipient mice were sensitized with OVA and complete Freund’s adjuvant (40 μg/animal). Spleen cells from non-sensitized **(D)** and sensitized mice **(E)** were obtained after 7 days and cultured in the presence of medium or *A. aegypti* SGE and stimulated with Con A (0.5 μg/mL) or OVA (100 μg/mL). Phenotype of naïve cells (CD62L^HIGH^/CD44^LOW^), T_EM_ subset (CD62L^LOW^ and CD44^HIGH^) and T_CM_ subset (CD62L^HIGH^ and CD44^HIGH^) from non-sensitized **(F)** or sensitized mice **(G)** were evaluated by flow cytometry after 72 h cultured in presence of medium or *A. aegypti* SGE and stimulated with Con A or OVA. ^*^p < 0.05 *versus* respective control group.

To further investigate the observed phenomenon, we generated memory cells by natural sensitization of mice to *A. aegypti* bites and evaluated the proliferation of spleen cells from these animals. As previously demonstrated (Figure 3D), *A. aegypti* SGE inhibits both basal cell metabolism and Con A-induced proliferation of spleen cells (Figure 5A). Contrarily, cells from mice sensitized with *A. aegypti* bites display antigen-specific proliferation in the presence of SGE (Figure 5B). Interestingly, when stimulated by Con A in the presence of SGE, proliferation of these cells was only partially inhibited, and the proportion of surviving cells corresponded to the amount of antigen specific proliferating cells (Figure 5B – dotted line). The secretion of neutralizing antibodies against salivary components by B lymphocytes present in the culture could not explain the lack of inhibition observed, since culture supernatants from spleen cells of sensitized mice, used as conditioned media, did not block (even partially) the effect of SGE on T cells from non-sensitized mice (Additional file 3).

We also investigated the effects of *A. aegypti* SGE on spleen cell proliferation of mice sensitized with bites of *An. aquasalis*, a species belonging to a different mosquito subfamily. Spleen cells from *An. aquasalis*-sensitized mice proliferate in the presence of SGE from this species, but not in the presence of *A. aegypti* SGE, indicating antigen-specific stimulation (Figure 5C). When these cells are incubated with both SGE (from *A. aegypti* and *An. aquasalis*), the proliferative response persists, confirming the lack of *A. aegypti* SGE activity on memory cells generated against *An. aquasalis* salivary components (Figure 5C). Additionally, when spleen cells from *An. aquasalis*-sensitized mice are stimulated with Con A or *An. aquasalis* SGE, a significant proliferation is observed, possibly from both naïve and memory cells. Stimulation with Con A in the presence of *A. aegypti* SGE only, or together with *An. aquasalis* SGE, induces a partial inhibition of the proliferative response. The partial blockage of proliferation observed suggests again that while the memory cell population survives and continues to proliferate, the naïve population dies in the culture (Figure 5C). This finding implies that *A. aegypti* SGE does not affect the memory cells generated against salivary components of other mosquito species (*e.g*. mice sensitized with *An. aquasalis* bites).

In order to further test our hypothesis, we generated memory cells against OVA, an antigen not related to mosquito salivary molecules. Recipient BALB/c mice were adoptively transferred with cells from DO11.10 mice and immunized with OVA to induce expansion of the donor cells and allow the generation of memory cells. One week later, spleen cells from recipient animals were preincubated *in vitro* with *A. aegypti* SGE and stimulated with Con A or OVA to assess the polyclonal and antigen-specific proliferation, respectively. Figure 5D shows that basal metabolism of cells incubated with medium or OVA, as well as Con A-induced proliferation from naïve mice are all significantly inhibited by *A. aegypti* SGE, as expected. On the contrary, spleen cells from mice adoptively transferred with DO11.10 cells and immunized with OVA had the Con A-induced proliferation only partially inhibited and, more important, antigen-specific proliferation was not affected at all (Figure 5E).

Finally, we evaluated the naïve and activated/memory markers in CD4^+^ T cell populations in medium only or under OVA stimulation. CD62L^HIGH^/CD44^LOW^ were considered naïve T cells while memory cells were divided into two subsets: effector memory T cells (T_EM_) that express CD62L^LOW^ and CD44^HIGH^; and central memory cells (T_CM_) which express CD62L^HIGH^ and CD44^HIGH^[31-35]. Figure 5 shows that both, T_EM_ and T_CM_ subsets, were increased in OVA-sensitized mice when compared to non-sensitized mice (upper left panels - Figures 5F and 5G). The addition of *A. aegypti* SGE to the cultures, affected naïve cell populations in both groups (lower left panels). The presence of OVA expanded both memory subsets in OVA-sensitized spleen cell cultures when compared to cultures of spleens from non-sensitized mice as expected (upper right panels – Figures 5F and 5G). Remarkably, coincubation with OVA plus SGE strongly affected CD62L^HIGH^/CD44^LOW^ naïve T cell population in both groups, while memory subsets were preserved or even increased, especially in the cultures from OVA-sensitized mice (lower right panels – Figures 5F and 5G). All these findings were concentration-dependent, as observed in Additional file 4. Taken together, these data show that naïve CD4^+^ T cells are susceptible, while memory T cells are selectively more resistant, to the apoptotic effect of *A. aegypti* SGE.

## Discussion

The blood-feeding behaviour is present in several orders of insects that have acquired the genetic and morphofunctional resources to suck, digest and use the blood of their vertebrate hosts [36]. Over millions of years of evolution, hematophagous mosquitoes have developed a complex pharmacological cocktail in their saliva, which clearly modulates host vascular and immune systems. Nevertheless, our knowledge of these processes is incomplete. The present study aimed to investigate the putative immunomodulatory effects of salivary components of *A. aegypti* mosquito vector on the differentiation, maturation and function of DCs and on the proliferation of T lymphocytes.

Pioneering work has shown that saliva of *Rhipicephalus sanguineus*, the “brown dog tick”, is able to inhibit differentiation and maturation of murine DCs [37]. In addition, Sá-Nunes *et al*. (2007) were the first to isolate and characterize prostaglandin E_2_ (PGE_2_) as the major DC modulator in saliva of *Ixodes scapularis* ticks, the Lyme disease vector [24]. Recently, PGE_2_ found in the saliva of *Dermacentor variabilis* ticks was also shown to regulate macrophage migration and cytokine production by these cells [38]. In addition, the presence of PGE_2_ in *R. sanguineus* saliva was also demonstrated, although in smaller amounts than *I. scapularis*[39]. However, the capacity of *R. sanguineus* saliva to modulate DCs is complemented by the presence of adenosine [39]. Additional studies have demonstrated the immunoregulatory and anti-inflammatory activity of crude tick saliva [40,41] and other proteinaceous components capable of modulating the function of DCs, such as Salp15 [42] and sialostatin L [27], both identified in the *I. scapularis* saliva. Although these previous pieces of evidence show a clear effect of the tick saliva on DCs, very little is known about the modulation of these cells by saliva of blood feeding insects. Costa *et al*. (2004) demonstrated that SGE of *Lutzomyia longipalpis* sandflies, one of the leishmaniasis vectors in the new world, affects cytokine production and costimulatory activity of human DCs [43]. Some years later, it was shown that SGE of *P. duboscqi* and *P. papatasi* sandfly species induced the production of PGE_2_ and IL-10 by DCs [44]. The observed effects of *P. papatasi* SGE on DCs was due to the presence of adenosine and adenosine monophosphate (5’ AMP) and this seems to be, at least partially, the mechanism by which the SGE of this species was able to decrease the arthritis symptoms in an autoimmune model induced by collagen [25].

DCs comprise distinct developmental and functional subsets present in lymphoid and non-lymphoid tissues and are involved in the activation of adaptive immune responses, but also in tolerance to self-antigens [45]. However, their frequencies in the tissues limit their experimental use. For example, Langerhans cells (the DC population from the epidermal layer of the skin) account for 3-5% of epidermal cells [46]. Accordingly, classical DCs such as those found in the dermis, represent 1-5% of total cells from peripheral tissues [45]. In addition, the increasing number of DC phenotypes described and isolation protocols employing enzymatic digestion which temporarily destroy surface markers, are other factors to consider [45]. Thus, although the BMDCs preparations employed in the present work do not precisely represent the population of epidermal and dermal DCs that possibly interact with mosquito saliva, the use of these cells to investigate the biological effects of salivary preparations or their purified components is accepted by most studies in the field [9,24,27,37,44,47]. To our surprise, *A. aegypti* SGE had no effect on DCs differentiation (Figure 1), maturation (Figure 2) and antigen presentation to T lymphocytes (Figure 3A). Corroborating with this data, it has been demonstrated that *A. aegypti* SGE did not affect the viability of a murine DC line [16]. These results contrast greatly with data described in other species of arthropod vectors and, in the specific case of *A. aegypti*, our results are original in demonstrating that direct modulation of DCs by salivary components does not seem to be a property of the saliva from this mosquito species.

Interestingly, addition of *A. aegypti* SGE to cultures of CD11c^+^ cells after washing caused a significant inhibition in antigen specific (Figure 3B) and polyclonal (Figure 3C) proliferation of CD4^+^ T lymphocytes. This data confirms that SGE acts directly on T lymphocytes and not on antigen-presenting cells, and corroborates with findings in the literature showing the negative modulation of lymphocyte function by *A. aegypti* salivary components [16-18]. We also observed that SGE of other insect species (namely *An. aquasalis*, and *P. duboscqi*) had no inhibitory effect on T cell proliferation (Figures 3E and 3F). Wanasen *et al*. [18] also observed the absence of effects on T cell proliferation employing SGE of *Culex quinquefasciatus*, which belongs to the same mosquito subfamily. To our knowledge, such inhibitory activity was only found in the SGE of another nematoceran species, the black fly *Simulium vittatum*[48].

We have also explored the mechanisms by which the *A. aegypti* SGE inhibits lymphocyte proliferation. Our results show that this inhibition occurs due to induction of apoptosis in spleen cells, more specifically on T (CD4^+^ and CD8^+^) and B (CD19^+^) cells (Figure 4A). The specificity of such biological activity is evidenced by DC assays, since differentiation, maturation and function were not affected, even when these cells were incubated with 40 μg/mL SGE, a 4-fold increase in the maximum concentration effect on the proliferative response. As previously reported, decreased T cell proliferation induced by *A. aegypti* SGE was due to diminished cell viability, as evaluated by PI and 7-AAD expression, both DNA markers [16,18]. It is important to emphasize that these markers are not specific for apoptotic cell death. Our findings unveiled that *A. aegypti* SGE induces apoptosis in T and B lymphocytes as assessed by exposure of phosphatidylserine (labeled with annexin V) at the surface of these cells (Figure 4A). Furthermore, our results suggest that caspase-3 (an executor caspase) and caspase-8 (an initiator caspase), but not Bim (a proapoptotic member of the intrinsic pathway), are likely to be involved in the apoptosis signaling induced by *A. aegypti* SGE (Figures 4B and 4C). As T and B cells are components of the adaptive immune response, it is reasonable to imagine that their effector functions (antibody secretion, cytotoxic granules or helper activities) are somehow deleterious to the mosquito life cycle. In fact, a classical study has demonstrated a decrease in the fecundity of mosquitoes fed on rabbits or guinea pigs immunized with a *A. aegypti* whole body homogenate [49].

Memory T cells are known to be more resistant to apoptosis than naïve T cells due to the increased expression of anti-apoptotic proteins [29]. Because cells from Bim^−/−^ mice are as susceptible to apoptosis as cells from Bim^+/−^ mice (Figure 4C), and it is well known that this differential resistance is considerably dependent on the neutralization of Bim-mediated apoptosis by increased levels of Bcl-2 [29,30], we investigated whether the *A. aegypti* salivary components would also have an effect on memory cells. Initially, mice were sensitized with *A. aegypti* bites and the effect of SGE on proliferative response of spleen cells from these animals was evaluated. Unlike naïve spleen cells (Figure 5A), those derived from sensitized animals proliferate in the presence of SGE (Figure 5B). In addition, when these cells were stimulated with Con A, only partial inhibition was achieved, suggesting that the memory cells present in the culture continue to proliferate even in the presence of SGE inhibitory factor(s). We rule out the role of neutralizing antibodies produced by B lymphocytes present in the culture on this effect, since spleen cell supernatants from sensitized mice, used as conditioned media, did not block the effect of SGE on T cells from non-sensitized mice (Additional file 3). Other explanations/mechanisms cannot be ruled out, such as peripheral tolerance and development of regulatory T cells, and will be explored in future work. We also demonstrated that SGE does not inhibit proliferation if memory cells are generated to other antigens. For example, spleen cells from *An. aquasalis*-sensitized mice proliferate when cultured with SGE of the same species, even in the presence of *A. aegypti* SGE. In addition, when these cells are co-cultured with Con A plus *A. aegypti* SGE, the inhibition of the proliferative response is only partial, reinforcing again our assumption that only naïve cells are affected by the presence of SGE in culture (Figure 5C).

This hypothesis is further supported by analyzing the proliferation of spleen cells from BALB/c mice receiving cells from a DO11.10 donor and subsequently immunized with OVA. When these cells are co-cultured with Con A plus SGE, the proliferation is partially inhibited (Figure 5B), whereas in cells from non-sensitized animals, SGE completely inhibits proliferation (Figure 5A). Remarkably, antigen-specific proliferation induced by OVA only occurs in sensitized mice, as expected, and it is not affected by *A. aegypti* SGE (Figure 5D and 5E). Regarding cell phenotype, SGE affects naïve cells (CD62L^HIGH^/CD44^LOW^) in both, control and immunized animals (Figure 5F and Figure 5G, respectively), while T_CM_ cells (CD62L^HIGH^/CD44^HIGH^) and T_EM_ cells (CD62L^LOW^/CD44^HIGH^) are proportionally more resistant in immunized animals (Figure 5G). So far, there is a single study showing a selective action of a salivary component in subpopulations of T cells. Such work demonstrated that Salp15, from the *I. scapularis* tick, binds to CD4 but not CD8 T cell co-receptor [50]. However, this is the first time that arthropod saliva has been shown to discriminate between naïve and memory T cells.

The blood feeding strategies greatly diverge between many hematophagous arthropods. While hard ticks maintain prolonged contact with host skin, some others, like mosquitoes and sandflies, are transient feeders and leave their host in minutes or even seconds. Undoubtedly, their strategies to modulate the host vascular and immune system may vary as well.

## Conclusions

Together, these results provide evidence for a complex interaction between *A. aegypti* salivary constituents and the host immune system. This pioneer study shows that saliva of a hematophagous arthropod is able to distinguish among different cell types (dendritic cells *versus* lymphocytes) and even subpopulations of the same cell type (naïve *versus* memory T cells). Whether this selectivity is important to mosquitoes feeding and reproduction remains to be determined. Therefore, the results generated by this work contributes to clarifying some of the features of the vector-host interaction providing a better understanding of the mechanisms used by the mosquito *A. aegypti* to circumvent the immune system of their hosts and successfully feed.

## Abbreviations

SGE: Salivary gland extract; FSDC: Fetal skin-derived DC line; BMDC: Bone marrow-derived dendritic cell; PGE2: Prostaglandin E_2_; 5’AMP: Adenosine monophosphate.

## Competing interests

The authors declare that they have no competing interests.

## Authors’ contributions

BB, MK, GPAM, EC, MLC and AS-N conceived the experimental design and assisted data interpretation. BB, MSB, CM, DIG, CNL, JC, MLC and AS-N carried out laboratory work. BB and AS-N wrote the manuscript’s draft. All authors read, reviewed and approved the final manuscript.

## Supplementary Material

Additional file 1 ***A. aegypti *** **SGE inhibits T cell proliferation.** BMDCs were pre-incubated with medium or 40 μg/mL of *A. aegypti* SGE overnight, washed 3 times and co-incubated with CD4^+^ from DO11.10 mice stained with CFSE. *A. aegypti* SGE was replaced in culture after washing and T cells were stimulated with Con A for 72 h. Cells were evaluated by flow cytometry as described in Methods. Click here for file

Additional file 2 ***A. aegypti *** **SGE induces changes in total spleen cell phenotype.** Indirect evaluation of the cell viability by examining the size (FSC) and internal complexity (SSC) of spleen cells incubated with Con A (B-G) for 72 h in the presence of increasing concentrations of *A. aegypti* SGE (0.1, 0.5, 1, 5 and 10 μg/mL) and compared with fresh cells (A). Click here for file

Additional file 3 **Antibodies produced by B lymphocytes do not neutralize SGE activity.** Three-day culture supernatants from different numbers of spleen cells from non-sensitized (A) and *A. aegypti*-sensitized (B) mice were used as a conditioned medium for cell cultures from a control mice spleen. These cells were then pre-incubated with medium alone or *A. aegypti* SGE and then stimulated with Con A for 72 h. Click here for file

Additional file 4 **Memory cells are resistant to *A. aegypti *** **SGE effects.** Non-adherent DO11.10 spleen cells were adoptively transferred to BALB/c mice and after 7 days, recipient mice were sensitized with OVA and complete Freund’s adjuvant (40 μg/animal). Spleen cells from non-sensitized and sensitized mice were obtained after 7 days and cultured in the presence of medium or *A. aegypti* SGE and stimulated with Con A (0.5 μg/mL) or OVA (100 μg/mL). Phenotype of naïve cells (CD62L^HIGH^/CD44^LOW^), T_EM_ subset (CD62L^LOW^ and CD44^HIGH^) and T_CM_ subset (CD62L^HIGH^ and CD44^HIGH^) from non-sensitized or sensitized mice were evaluated by flow cytometry after 72 h cultured in presence of medium or *A. aegypti* SGE and stimulated with Con A or OVA. Click here for file
