# Supplementary figures and images for: Effects of Aedes aegypti salivary components on dendritic cell and lymphocyte biology
Source: Parasit Vectors. 2013 Nov 15;6:329. doi: 10.1186/1756-3305-6-329 (PMC3843549; doi:10.1186/1756-3305-6-329)

Additional file 1

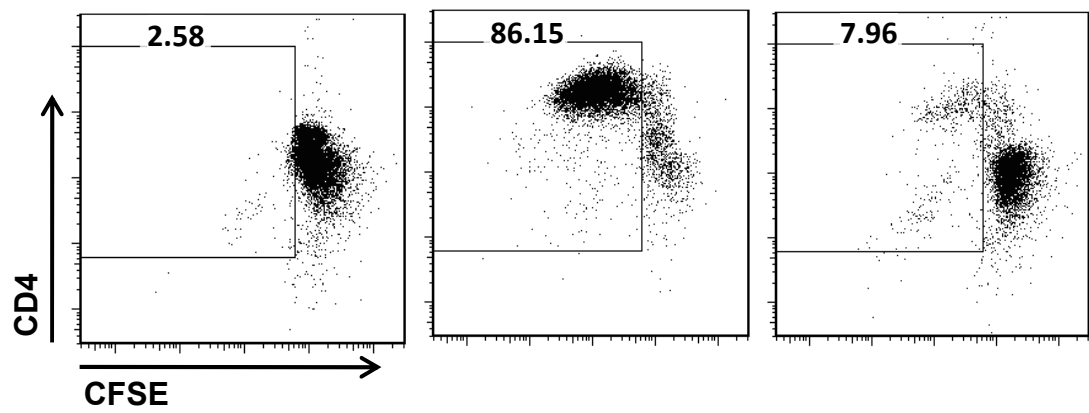

|                   |   |   |   |
|-------------------|---|---|---|
| SGE (40 µg/mL)    | - | - | + |
| Con A (0.5 µg/mL) | - | + | + |

Supplement: Additional file 1 — A. aegypti SGE inhibits T cell proliferation. BMDCs were pre-incubated with medium or 40 μg/mL of A. aegypti SGE overnight, washed 3 times and co-incubated with CD4+ from DO11.10 mice stained with CFSE. A. aegypti SGE was replaced in culture after washing and T cells were stimulated with Con A for 72 h. Cells were evaluated by flow cytometry as described in Methods. [file 1756-3305-6-329-S1.pdf]

## Additional file 2

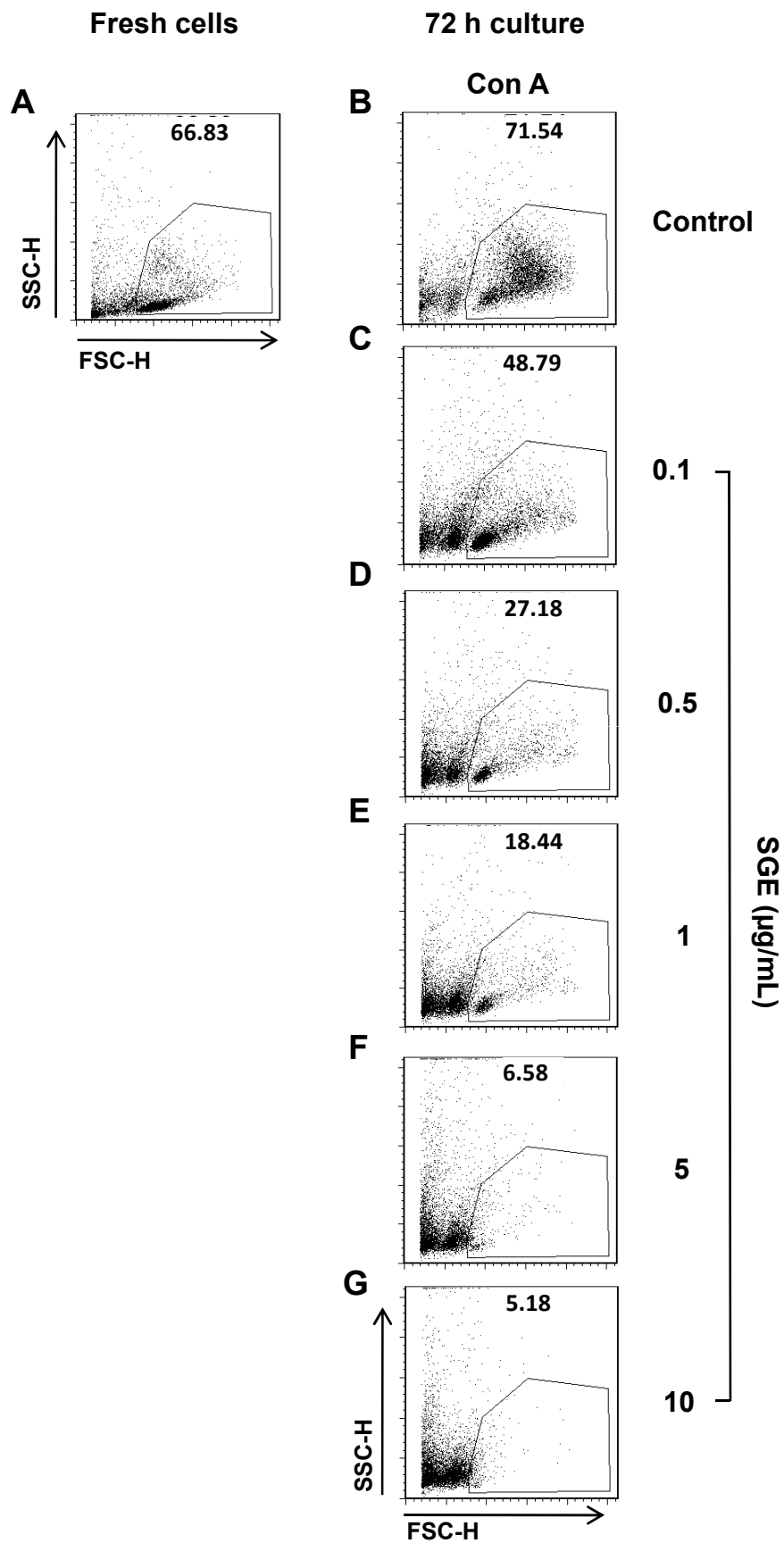

Supplement: Additional file 2 — A. aegypti SGE induces changes in total spleen cell phenotype. Indirect evaluation of the cell viability by examining the size (FSC) and internal complexity (SSC) of spleen cells incubated with Con A (B-G) for 72 h in the presence of increasing concentrations of A. aegypti SGE (0.1, 0.5, 1, 5 and 10 μg/mL) and compared with fresh cells (A). [file 1756-3305-6-329-S2.pdf]

Additional file 3

A

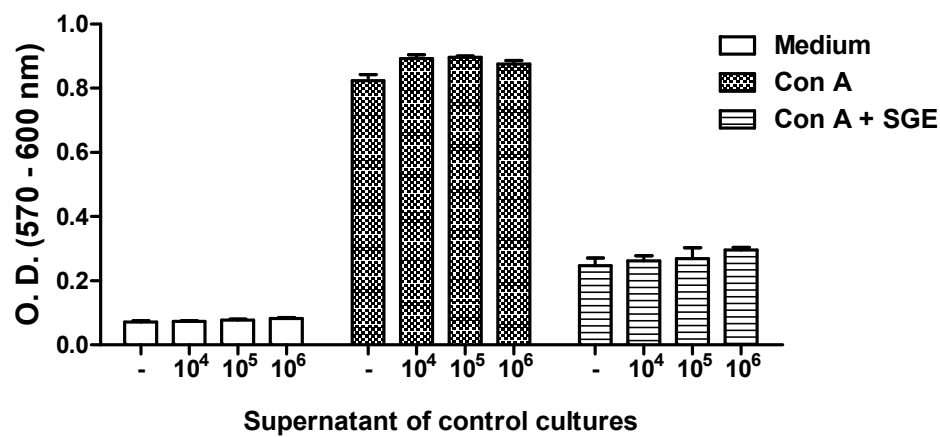

B

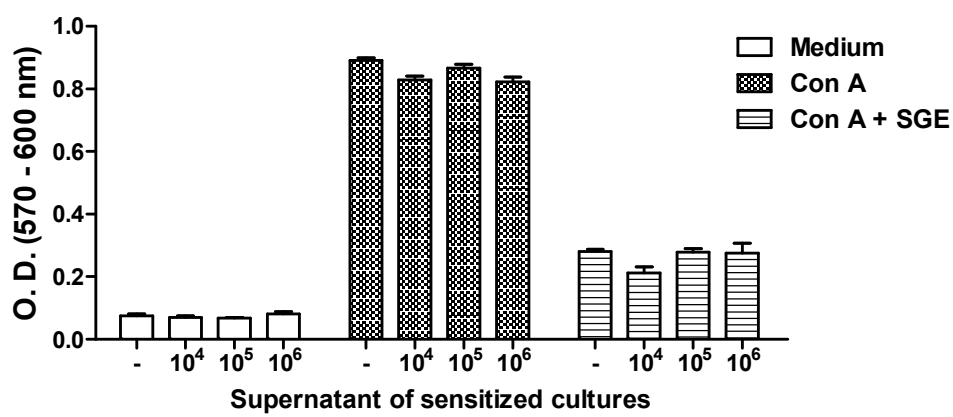

Supplement: Additional file 3 — Antibodies produced by B lymphocytes do not neutralize SGE activity. Three-day culture supernatants from different numbers of spleen cells from non-sensitized (A) and A. aegypti-sensitized (B) mice were used as a conditioned medium for cell cultures from a control mice spleen. These cells were then pre-incubated with medium alone or A. aegypti SGE and then stimulated with Con A for 72 h. [file 1756-3305-6-329-S3.pdf]

Additional file 4

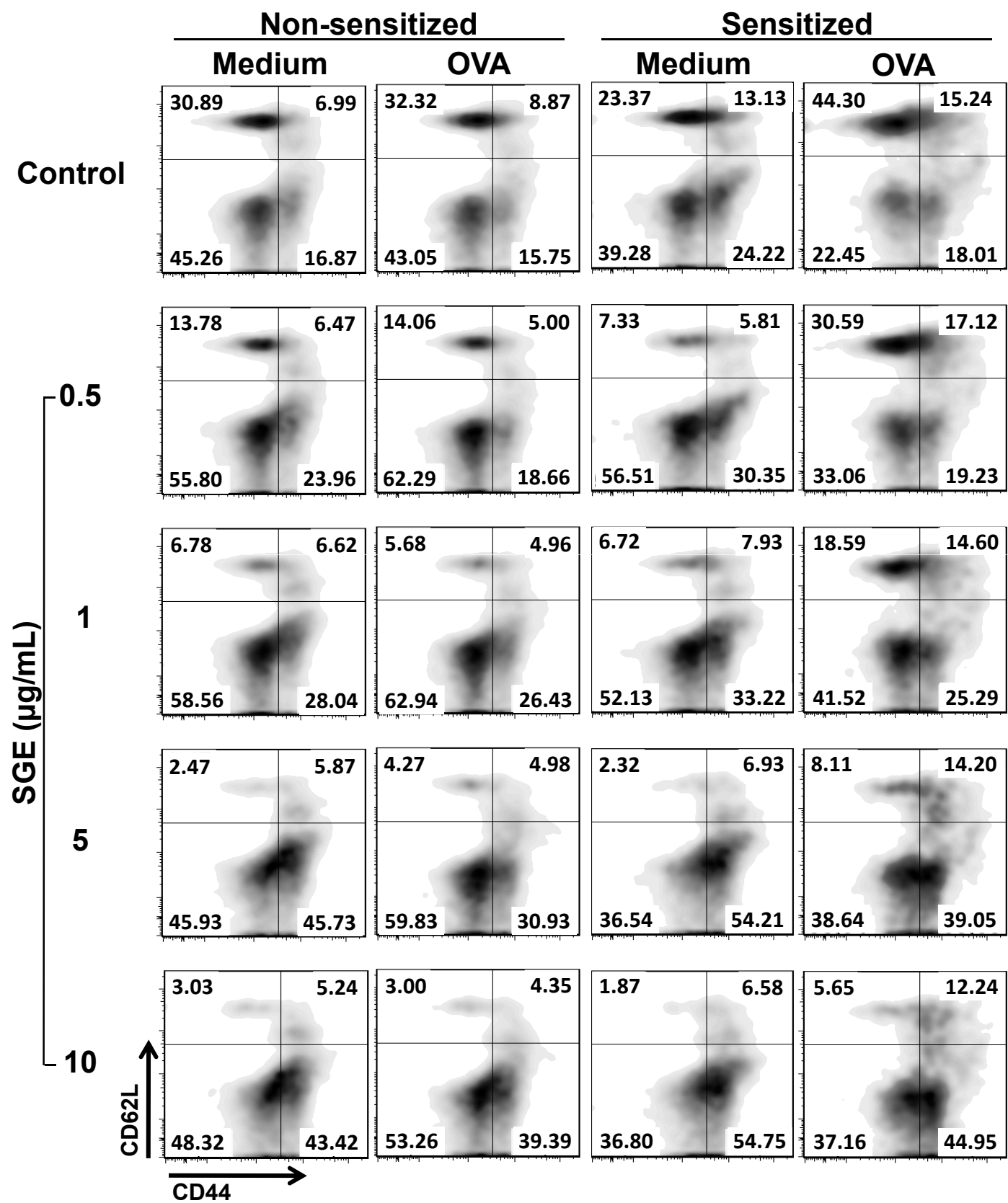

Supplement: Additional file 4 — Memory cells are resistant to A. aegypti SGE effects. Non-adherent DO11.10 spleen cells were adoptively transferred to BALB/c mice and after 7 days, recipient mice were sensitized with OVA and complete Freund’s adjuvant (40 μg/animal). Spleen cells from non-sensitized and sensitized mice were obtained after 7 days and cultured in the presence of medium or A. aegypti SGE and stimulated with Con A (0.5 μg/mL) or OVA (100 μg/mL). Phenotype of naïve cells (CD62LHIGH/CD44LOW), TEM subset (CD62LLOW and CD44HIGH) and TCM subset (CD62LHIGH and CD44HIGH) from non-sensitized or sensitized mice were evaluated by flow cytometry after 72 h cultured in presence of medium or A. aegypti SGE and stimulated with Con A or OVA. [file 1756-3305-6-329-S4.pdf]
